# SARS-CoV-2 RBD Scaffolded by AP205 or TIP60 Nanoparticles and Delivered as mRNA Elicits Robust Neutralizing Antibody Responses

**DOI:** 10.3390/vaccines13080778

**Published:** 2025-07-22

**Authors:** Johnathan D. Guest, Yi Zhang, Daniel Flores, Emily Atkins, Kuishu Ren, Yingyun Cai, Kim Rosenthal, Zimeng Wang, Kihwan Kim, Charles Chen, Richard Roque, Bei Cheng, Marianna Yanez Arteta, Liping Zhou, Jason Laliberte, Joseph R. Francica

**Affiliations:** 1 Vaccines and Immune Therapies, AstraZeneca, Gaithersburg, MD 20878, USA; johnathan.guest@astrazeneca.com (J.D.G.); yi.zhang29@astrazeneca.com (Y.Z.); daniel.flores@astrazeneca.com (D.F.); emily.atkins@astrazeneca.com (E.A.); kuishu.ren@astrazeneca.com (K.R.); yingyun.cai@astrazeneca.com (Y.C.); kim.rosenthal@astrazeneca.com (K.R.); kihwan.kim@astrazeneca.com (K.K.); richard.roque@astrazeneca.com (R.R.); jason.laliberte@astrazeneca.com (J.L.); 2 Advanced Drug Delivery, Pharmaceutical Sciences, AstraZeneca, Waltham, MA 02451, USA; zimeng.wang@astrazeneca.com (Z.W.); charles.chen1@astrazeneca.com (C.C.); bei.cheng2@astrazeneca.com (B.C.); liping.zhou1@astrazeneca.com (L.Z.); 3 Advanced Drug Delivery, Pharmaceutical Sciences, AstraZeneca, 43183 Gothenburg, Sweden; mariann.yanezarteta@astrazeneca.com

**Keywords:** SARS-CoV-2, mRNA vaccines, protein scaffold, nanoparticles, antibody neutralization

## Abstract

Background/Objectives: SARS-CoV-2 vaccine candidates comprising the receptor binding domain (RBD) of the spike protein have been shown to confer protection against infection. Previous research evaluating vaccine candidates with SARS-CoV-2 RBD fused to ferritin (RBD-ferritin) and other scaffolds suggested that multimeric assemblies of RBD can enhance antigen presentation to improve the potency and breadth of immune responses. Though RBDs directly fused to a self-assembling scaffold can be delivered as messenger RNA (mRNA) formulated with lipid nanoparticles (LNPs), reports of SARS-CoV-2 vaccine candidates that combine these approaches remain scarce. Methods: Here, we designed RBD fused to AP205 or TIP60 self-assembling nanoparticles following a search of available structures focused on several scaffold properties. RBD-AP205 and RBD-TIP60 were tested for antigenicity following transfection and for immunogenicity and neutralization potency when delivered as mRNA in mice, with RBD-ferritin as a direct comparator. Results: All scaffolded RBD constructs were readily secreted to transfection supernatant and showed antigenicity in ELISA, though clear heterogeneity in assembly was observed. RBD-AP205 and RBD-TIP60 also exhibited robust antibody binding and neutralization titers in mice that were comparable to those elicited by RBD-ferritin or a full-length membrane-bound spike. Conclusions: These data suggest that AP205 and TIP60 can present RBD as effectively as ferritin and induce similar immune responses. By describing additional scaffolds for multimeric display that accommodate mRNA delivery platforms, this work can provide new tools for future vaccine design efforts.

## 1. Introduction

Severe acute respiratory syndrome coronavirus 2 (SARS-CoV-2) emerged in late 2019, becoming a global pandemic that continues to cause viral infections, hospitalizations, and deaths [1]. Several vaccines have been licensed, utilizing mRNA [2,3], adenovirus [4], and recombinant protein [5] technologies, with seasonal boosters to accommodate new variants. These vaccines incorporate the full-length SARS-CoV-2 spike as their antigen, driving potent immunogenicity and neutralizing responses that protect from severe illness. A crucial domain within the spike protein is the receptor binding domain (RBD), which enables viral entry and infection through interactions with its receptor, ACE2 [6,7]. Numerous monoclonal antibodies targeting RBD have been found to potently neutralize SARS-CoV-2 by blocking ACE2 recognition [8,9,10]. However, Omicron variants have accumulated several dozen RBD mutations, helping the virus evade RBD antibodies that can bind and neutralize ancestral variants [11,12,13,14]. Other RBD epitopes are more conserved and can be targeted by broad neutralizing antibodies that do not block ACE2, but these antibodies are often rarer and less potent [15,16]. New immunogen designs maximizing the presentation of RBD epitopes, especially those that can induce cross-neutralizing antibodies, could potentially improve future SARS-CoV-2 vaccines.

As a result, several vaccine design efforts have focused on immunization with SARS-CoV-2 RBD rather than full spike, to focus immune responses on this critical domain [17]. While several vaccine candidates presenting monomeric, dimeric, or trimeric RBD have been explored [18,19,20,21,22,23], other research has utilized multimeric self-assembling nanoparticles, or scaffolds, to present an ordered array of the RBD on a protein surface [24,25,26,27,28]. This multimerization has been found to increase the immunogenicity of protein subunits and trimeric antigens of multiple viruses, as a repetitive and highly ordered display of an antigen likely induces greater B-cell activation and crosslinking [29,30,31,32]. Many designs have directly fused RBD to a scaffold of interest with a short and flexible linker, facilitating self-assembly from RBD-scaffold monomers to particles that present RBD subunits. Other linking mechanisms for scaffolding RBD have included chemical conjugation [33,34], SpyTag/SpyCatcher conjugation [35,36], enzymatic conjugation [37], or Fc-protein A binding [38] to a nanoparticle surface. However, these methods require multiple steps to achieve full assembly after protein purification, making them incompatible with mRNA vaccines delivered via lipid nanoparticles (LNPs).

To combine the concepts of vaccine nanoparticles with mRNA delivery, several vaccine scaffolds such as *Helicobacter pylori* (*H. pylori*) ferritin and lumazine synthase from *Aquifex aeolicus* have been directly fused to SARS-CoV-2 receptor binding domain (RBD) subunits [24,39,40,41,42,43,44] and encoded as mRNA for vaccination [45,46,47]. *H. pylori* ferritin represents a 24-mer assembly found in many different organisms [48,49] and whose self-assembling particle properties have been applied to multiple vaccine design efforts [50,51]. While ferritin-scaffolded designs have shown to be immunogenic, intact ferritin particles are relatively small (~10 nm in diameter) compared to other large structurally characterized assemblies. Because of ferritin’s size, these designs may not be optimal for inducing B-cell responses, as larger particles tend to stimulate more crosslinking and signaling [52,53], and would be more comparable in size to infectious SARS-CoV-2 viral particles [54]. At the same time, the antigen spacing inherent to presentation with ferritin could be replicated by other scaffolds that either provide more potential space between subunits due to their larger size or already present evenly spaced attachment points on the surface. Some research has already investigated how the precise control of antigen spacing can affect immune responses [55,56], yet the influence of the presentation of the same antigen by different scaffolds is unclear. Lastly, the number of scaffolded RBD constructs tested as mRNA-LNPs remains limited; testing more large particles directly fused to RBD in this delivery platform could help fill the gap in knowledge regarding the use of self-assembling scaffolds as mRNA.

Therefore, with RBD-ferritin as a benchmark [57], we sought to explore the use of other large assemblies AP205, a phage coat protein, and TIP60, a designed icosahedral nanoparticle, as vaccine scaffolds to present SARS-CoV-2 RBD subunits. Mimicking the RBD-ferritin construct, the C-terminus of RBD was fused to the N-terminus of AP205 or TIP60. This research studied both their antigenicity and immunogenicity when delivered as mRNA-LNPs to better understand how changes in scaffold display affect immune responses to the SARS-CoV-2 RBD [8,22,55]. Through comparisons to established vaccine candidates, this study identifies additional nanoparticles that may be useful for the scaffolding of protein subunits.

## 2. Methods

### 2.1. Structural Modeling and Analysis

Design work began with a search of experimentally determined nanoparticles based on several properties as follows. Nanoparticle assemblies in RCSB Protein Data Bank (PDB) (RSCB.org, accessed 1 September 2022) were filtered for (1) structures with icosahedral or tetrahedral symmetry, (2) sequence length < 300 amino acids to accommodate mRNA transcript constraints, and (3) only those of non-eukaryotic or synthetic origin were considered to avoid autoreactivity if developed for human use. Finally, exposure of N-termini to the surface of the particle was confirmed through visualization of the assembly in PyMOL (Schrodinger). This search resulted in the selection of AP205 and TIP60 for further study.

Model assemblies of RBD-ferritin, RBD-TIP60, and RBD-AP205 were constructed using homology modeling in Rosetta and visualization with PyMOL (Schrodinger). The following structures were obtained from RCSB PDB (RCSB.org) and used as templates: BA.4/5 RBD—PDB code 7ZXU [58], AP205—PDB code 5LQP [59], TIP60—PDB code 7EQ9 [60], *H. pylori* ferritin—PDB code 3EGM [61], and bullfrog ferritin extension—PDB code 1RCC [62]. Briefly, an RBD structure was connected to each scaffold by modeling a glycine–serine linker (GSGGGG) in between the monomers after positioning them close together in 3D space, then using either RosettaCM [63] or Rosetta Remodel [64] for homology modeling. Monomer models were then copied and aligned to the full assembly of each scaffold. Measurements of distance in angstroms between N-terminal attachment points and to estimate the diameter of model assemblies were conducted using the measurement wizard in PyMOL.

### 2.2. Gibson Cloning and Assembly

All designs were cloned using a RBD-ferritin plasmid as a template. A map of the RBD-ferritin template plasmid is shown in Figure S1. For RBD-AP205 and RBD-TIP60 cloning, we replaced the ferritin sequence using Gibson assembly with primers to open the plasmid vector and inserted a codon-optimized gene fragment for the AP205 or TIP60 scaffold [65]. Synthesized gene fragments and accompanying primers were ordered from IDT (Coralville, IA, USA). The primer sequences are as follows:

- RBD-AP205 F: 5′—GTCTCCTCCGATACAACTTGATGATAAGTCTAGAGGGC—3′
- RBD-AP205 R: 5′—GGCTGCATGGGCTTATTGCCCCCTCCACCAGAACC—3′
- RBD-TIP60 F: 5′—CAGACGCCTTGAGGAGGAATGATGATAAGTCTAGAGGG—3′
- RBD-TIP60 R: 5′—CATTATTTTTATATTTTTGCCCCCTCCACCAGAACC—3′

Following PCR amplification of the vector, AP205 and TIP60 fragments were used to replace the scaffold sequence in the RBD-ferritin plasmid template. Assembled clones were transformed in 10β cells (NEB, Ipswich, MA, USA). DNA was isolated by a spin miniprep kit (Qiagen, Hilden, Germany) and confirmed by Sanger sequencing (Psomagen, Rockville, MD, USA).

### 2.3. Transient Transfection

For testing the small-scale expression of designs, endotoxin-free maxiprep DNA was used to transfect 30 mL of HEK293F cells at a density of 1 × 10^6^ cells/mL in Freestyle 293 media (Gibco, Paisley, UK) [66,67]. Maxiprep DNA was obtained using a PureLink HiPure Plasmid Maxiprep Kit (ThermoFisher, Vilnius, Lithuania). 293fectin (Gibco, Carlsbad, CA, USA) diluted in OptiMEM (ThermoFisher, Grand Island, NY, USA) was mixed 1:1 with DNA diluted in OptiMEM. A mock transfection was also conducted, in which only OptiMEM was added to cells. Transfections were incubated at 37 °C with 8% CO_2_ shaking at 125 rpm for 4–5 days, after which the supernatant was harvested and clarified.

### 2.4. Enzyme-Linked Immunosorbent Assay (ELISA)

Supernatant samples from transfection were tested in a sandwich ELISA for binding to a pair of RBD antibodies that bind to non-overlapping epitopes [68,69,70]. First, an anti-RBD antibody engineered with a mouse Fc antibody region (AZ internal reagent, Gaithersburg, MD, USA) was coated on 384-well ELISA plates at 5 µg/mL and incubated overnight in 4 °C. Dilutions of transfected supernatant were prepared in three technical replicates starting with neat supernatant, followed by 5-fold serial dilutions with casein in PBS (ThermoFisher, Rockford, IL, USA). After incubation with casein as a blocking buffer, supernatant dilutions were incubated on the plate for 2 h at 37 °C. Anti-RBD antibody Ly-CoV1404 [71] was diluted to 3 µg/mL in casein and used as the primary antibody, followed by goat anti-Human IgG HRP (ThermoFisher, Rockford, IL, USA) diluted 1:10,000 in PBS Tween (0.1%) as the secondary antibody.

To measure endpoint titers, 384-well MaxiSorp plates (ThermoFisher, Rochester, NY, USA) were coated with 5 µg/mL of SARS-CoV-2 RBD or 3 µg/mL of betacoronavirus spike antigen and incubated overnight at 4 °C. RBD proteins of Omicron variants (BA.4/5 and XBB.1.5) and ancestral WT were purified in-house, while other betacoronavirus spike proteins were ordered commercially and resuspended from lyophilization (ACROBiosystems, Newark, DE, USA). The tested betacoronavirus spike proteins included SARS-CoV (accession # AAP13567.1), HKU3 (accession # Q3LZX1), BtKY72 (accession # APO40579.1), and MERS-CoV (accession # YP_009047204.1). The next day, ELISA plates were incubated with blocking buffer (casein in PBS) at RT. Two weeks post 1st dose (2wp1), two weeks post 2nd dose (2wp2), or four weeks post 2nd dose (4wp2) sera were thawed and diluted in a 96-well plate format, with a 1:50 starting dilution and 5-fold serial dilutions in casein buffer. Diluted sera were added to each ELISA plate and incubated at RT with shaking. Plates were washed with PBS Tween (0.1%), patted dry, then incubated with anti-mouse IgG HRP diluted 1:10,000 as a secondary antibody (Dako, Santa Clara, CA, USA).

For all assays, plates were washed with PBS Tween after a 30 min incubation with secondary antibody at RT. 3,3′,5,5′-Tetramethylbenzidine (TMB) substrate (SeraCare, Milford, MA, USA) was added to all wells, then plates were sealed and incubated at RT in the dark for 5 min. Subsequently, 1 N sulfuric acid was added immediately to stop the reaction. Plates were then read by absorbance at 450 nm on an EnVision plate reader (PerkinElmer, Waltham, MA, USA) in a 384-well plate format. Dilution curves were used to calculate endpoint titers in Prism, and overall endpoint titers for each group were calculated as a geometric mean.

### 2.5. Gel Electrophoresis, Coomassie Staining, and Western Blot

Harvested supernatants were concentrated at 14,000× *g* with Amicon ultra-centrifugal filters (Sigma-Aldrich, Cork, Ireland). For SDS-PAGE [72], supernatant and purified protein samples were loaded in a 4–12% Bis-tris NuPAGE gel according to an A280 value measured in NanoDrop One spectrophotometer (ThermoFisher, Wilmington, DE, USA). Each sample was incubated with LDS sample buffer (Invitrogen) and sample reducing agent (Invitrogen, Carlsbad, CA, USA), then heated at 95 °C for 5 min to complete denaturation. Bis-tris gels were run for 35 min at 200 volts in 1× MES buffer (Invitrogen, Carlsbad, CA, USA). For NativePAGE [73], supernatant and purified protein samples were loaded in a 3–8% Tris-Acetate gel (ThermoFisher, Carlsbad, CA, USA) according to measured A280. Each sample was incubated with native Tris-Glycine sample buffer (Novex, Carlsbad, CA, USA) and run for 3 h at 150 volts in 1× Tris-glycine native running buffer (ThermoFisher, Carlsbad, CA, USA). All gel electrophoresis was conducted in a XCell SureLock electrophoresis cell (Invitrogen, Carlsbad, CA, USA) with a 300 V Electrophoresis Power Supply (Avantor, Phillipsburg, NJ, USA). For coomassie blue staining [74], extracted gels were incubated with InstantBlue Protein Stain (Novus Biologicals, Centennial, CO, USA) at RT with shaking for at least two hours. To destain, the stained gel was incubated with USP water twice for 1–2 h. For western blot [75], SDS-PAGE gels were first transferred using an iBlot 2 PVDF mini stack in the iBlot2 system (Invitrogen, Kiryat Shmona, Israel) at 15 volts for 7 min. After blocking each membrane with 5% bovine serum albumin (BSA) in PBS, anti-RBD HL257 monoclonal antibody (Invitrogen, Rockford, IL, USA) was diluted 1:5000 (0.2 µg/mL) in PBS Tween (0.1%) and used as the primary antibody. Goat anti-rabbit IgG conjugated with horseradish peroxidase (Jackson ImmunoResearch, West Grove, PA, USA) was diluted 1:10,000 and used as the secondary antibody. SuperSignal West Pico PLUS chemiluminescent substrate (ThermoFisher, Rockford, IL, USA) was mixed 1:1 and added to the membrane prior to imaging. All gel images were obtained using the ChemiDoc system (BioRad, Hercules, CA, USA). The following markers were used to determine the molecular weight of bands: MagicMark XP (Invitrogen, Carlsbad, CA, USA) for the western blot under reducing conditions, HiMark Pre-stained (Invitrogen, Carlsbad, CA, USA) for the western blot under native conditions, and NativeMark Unstained (Invitrogen, Carlsbad, CA, USA) for coomassie blue under native conditions. Following transfer to a PVDF membrane, the ladder bands in the western blot under native conditions were marked with a WesternBright ChemiPen (Advansta, San Jose, CA, USA) to allow for detection. The molecular weights of RBD-AP205, RBD-TIP60, and RBD-ferritin monomers were estimated using the Expasy ProtParam server [76], with 2 kDa added per N-linked glycosylation site through an approximation within the molecular weight range of previously identified glycans [77]. The molecular weight estimations for monomers were as follows: RBD-AP205—44 kDa, RBD-TIP60—45 kDa, RBD-ferritin—47 kDa. Image Lab software (BioRad, Hercules, CA, USA) was utilized both for calculating intensity ratios for western blot bands relative to background intensity and for coloration of the coomassie blue gel in Figure S3B.

### 2.6. In Vitro Transcription (IVT)

To produce messenger RNA (mRNA) [78,79,80], the coding sequence of each scaffold design was first cloned in a pMRNA vector with a polyA tail flanked by a BspQI enzyme restriction site. Following transformation, DNA was isolated by miniprep kit (Qiagen) and confirmed by Sanger sequencing (Psomagen, Rockville, MD, USA). Endotoxin-free maxiprep DNA was isolated from 100–150 mL bacterial cultures using the ZymoPURE II Maxiprep kit (Zymogen, Irvine, CA, USA). For DNA linearization, 20 μg of maxiprep DNA was incubated with 2.5 μL of 10,000 units/mL BspQI enzyme (NEB, Ipswich, MA, USA) at 50 °C for 2–4 h. Digest reactions were mixed 1:1 with phenol/chloroform/isoamyl alcohol (25:24:1 ratio (*v*/*v*); Invitrogen, Carlsbad, CA, USA), then chloroform/isoamyl alcohol (24:1 ratio (*v*/*v*); Arcos, Kiryat Shmona, Israel), with centrifugation and removal of the aqueous layer between mixing steps. Linearized DNA was then isolated using 3M sodium acetate pH 5.5 (Invitrogen, Vilnius, Lithuania) and 100% ethanol (Sigma-Aldrich, Sheboygan Falls, WI, USA), and the isolated DNA was washed with 70% ethanol after incubation at −20 °C. After centrifugation and removal of supernatant, DNA was resuspended in water, and the purified DNA concentration was measured by NanoDrop.

IVT was conducted using the Hiscribe T7 High Yield RNA Synthesis Kit (NEB, Ipswich, MA, USA), with each reaction conducted for 5–10 µg of linearized DNA. N1-methyl-pseudouridine (TriLink, San Diego, CA, USA) was used in each IVT reaction instead of the UTP supplied by the kit. CleanCap AG (100 mM concentration; TriLink, San Diego, CA, USA) was also added to each reaction at this stage, comprising 1/10^th^ of the total reaction volume. IVT reactions were incubated at 37 °C for 5 h, then mixed with DnaseI (NEB, Ipswich, MA, USA), 10× DnaseI buffer, and water to reduce viscosity. After incubation at 37 °C for 15 min, cold lithium chloride (Invitrogen, Vilnius, Lithuania) was added to a final concentration of 2.5 M. The mixture was then incubated at −20 °C for at least 30 min. After several steps of centrifugation, removing supernatant, and washing with cold 70% ethanol, the remaining pellet was resuspended in RNA storage solution (ThermoFisher, Vilnius, Lithuania) and incubated on ice in 4 °C until pellet resuspension was complete. The concentration of RNA was measured by Nanodrop, then RNA aliquots were stored at −80 °C. RNA Screentape and Tapestation system (Agilent, Santa Clara, CA, USA) were used to check the quality of mRNA diluted to linear range of 50–500 ng/µL prior to LNP formulations.

### 2.7. Formulation of mRNA Lipid Nanoparticles (LNPs)

The LNP formulations were prepared using a modified procedure of a microfluidic method previously described [81,82]. The stock solution of lipids is as follows: cholesterol, distearoylphosphatidylcholine (DSPC), and 1,2-Dimyristoyl-sn-Glycero-3-Phosphoethanolamine-N-[methoxy(polyethylene glycol)-2000] (DMPE-PEG 2000) lipids were each dissolved in ethanol at 20 mg/mL, while the ionizable cationic lipid (AZ internal reagent) was dissolved in ethanol at 40 mg/mL. Lipid dissolution was aided through incubation in water at 50 °C and/or vortexing. Lipid solutions were ready to use once the resulting solutions were clear. The ionizable lipid, cholesterol, DSPC, and DMPE-PEG 2000 were mixed at the molar ratio of 50:38.5:10:1.5, resulting in a total molar concentration of 12.5 mM for LNP fabrication. Frozen mRNA was thawed first on ice then brought to room temperature (RT). The mRNA stock solution was mixed thoroughly with nuclease free water followed by a 100 mM citrate buffer to achieve a final citrate concentration of 50 mM at pH of 3. In the resulting solution, the final RNA concentration was 0.122 mg/mL. After mixing, the RNA/citrate solution was used immediately to prevent RNA degradation or hydrolysis. With an ionizable lipid nitrogen/oligonucleotide phosphate (N/P) molar ratio of 6, the mRNA solution was combined with the lipid mixture at a volume ratio of 3:1 (mRNA in aqueous: lipids in ethanol) via a NanoAssemblr Ignite microfluidic system (Precision Nanosystems, Vancouver, Canada) at a flow rate of 12 mL/min. Filtered LNPs were dialyzed in Slide-A-Lyzer dialysis cassettes (ThermoFisher, Rockford, IL, USA), first against 1× PBS (pH 7.4) overnight and then against 20 mM Tris HCl buffer (pH 7.4) overnight. Crude LNPs were filtered through 0.22 µm Acrodisc^®^ Syringe Filters with Supor^®^ Membrane (PALL, Fajardo, Puerto Rico). Dialyzed LNPs were concentrated using Amicon ultra-centrifugal filters (Sigma-Aldrich, Cork, Ireland) at 3000 rpm to reach a target concentration. Concentrated LNPs were mixed with 40% sucrose in 20 mM Tris HCl (pH 7.4). The final LNP suspension was at 0.2 mg/mL mRNA concentration in 20 mM Tris-8% sucrose (pH = 7.4) and stored at −80 °C until use.

Formulations were tested for particle size, polydispersity index (PDI), RNA encapsulation, and endotoxin concentration. Size and PDI of LNPs were determined by dynamic light scattering (DLS) measurements using a Zetasizer (Malvern Panalytical, Malvern, UK). mRNA concentration and encapsulation efficiency in LNP were determined using the RiboGreen assay with the Quan-it kit (ThermoFisher, Carlsbad, CA, USA). To test endotoxin concentrations, mRNA-LNP suspensions were first thawed and diluted 1:100 in PBS to 2 µg/mL. A total of 25 µl of diluted sample was then loaded into each well of an EndoSafe Limulus Amebocyte Lysate (LAL) Cartridge (Charles River, Charleston, SC, USA) and evaluated using an EndoSafe Nextgen-PTS spectrophotometer (Charles River, Charleston, SC, USA) with a standard curve of 5 EU/mL to 0.05 EU/mL). Endotoxin concentrations were initially reported as EU/µg, which were used to calculate EU/mL according to the dilution factor.

### 2.8. In Vivo Methods

SARS-CoV-2 RBD and spike vaccine antibody response was assessed through an immunogenicity study in mice. This study was approved by the IACUC (protocol number AUP-22-49) and conducted at AstraZeneca, PLC, in adherence of the following standards of the Association for Assessment and Accreditation of Laboratory Animal Care: the 8th edition of the Guide for the Care and Use of Laboratory Animals; the Animal Welfare Act; and the 2015 reprint of the Public Health Service Policy on Human Care and Use of Laboratory Animals. The total number of animals used, group sizes, and number of groups were considered the minimum required to properly characterize the effects of SARS-CoV-2 scaffolded RBD designs, full-length membrane-bound (FLMB) spike, and the PBS control. The study was designed so that it did not need an unnecessary number of animals to accomplish its objectives, and animals were randomly assigned into their respective treatment groups. Personnel who performed the immunization were not blinded to treatment groups. Reporting of In Vivo Experiments (ARRIVE, 2.0) guidelines were also followed regarding the performance of all animal studies. Interventions to reduce pain, suffering, and distress were taken whenever possible in accordance with these protocols.

Female BALB/c mice were sourced from Jackson Laboratories and ranged between 5–7 weeks old upon arrival. Each treatment animal (six per group) received an intramuscular injection of 1 μg mRNA-LNP three weeks apart on Day 0 and Day 21; three control animals received PBS alone at equivalent volumes as the treatment animals. All injections were administered in the left gracilis in 50 µl. Blood samples were collected via the submandibular vein using a cheek punch technique at various times up to four weeks post 2nd dose to measure serum antibody concentrations. At study termination, animals were euthanized by carbon dioxide asphyxiation, tested for response to painful stimuli followed by exsanguination via cardiac puncture. The study design is summarized in the results section.

### 2.9. SARS-CoV-2 Pseudovirus Neutralization Assay

SARS-CoV-2 pseudoviruses were generated as previously described [83]. Briefly, we utilized a lentiviral vector system in which Freestyle 293X cells were co-transfected with lentiviral packaging plasmids, a luciferase reporter plasmid, and a SARS-CoV-2 spike plasmid. Separate plasmids were used to generate pseudoviruses bearing the spike of Omicron BA.4/5, XBB.1.5, and BQ.1.1 variants. The viral supernatant was harvested 48 h later, clarified through low-speed centrifugation and filtration, and purified through centrifugation with a 10% sucrose cushion at a 4:1 *v*/*v* ratio of virus:sucrose. Serial dilutions of mouse serum were prepared in a 96-well microtiter plate and pre-incubated with pseudovirus for 60 min at 37 °C, to which Ad293 cells that stably express ACE2 were added. The plate was returned to the 37 °C incubator for 48 h and luciferase activity was measured on an EnVision 2105 Multimode Plate Reader (PerkinElmer, Waltham, MA, USA) using the Steady Glo Luciferase Assay System (Promega, Madison, WI, USA) according to the manufacturer’s recommendations. Half-maximal inhibitory dilutions (ID_50_) were determined from nonlinear regression analyses.

### 2.10. Graphing and Statistical Tests

All ELISA and neutralization plots were created in Prism version 10.4.1 (Graphpad). Quantification of statistically significant differences between groups was determined by the Kruskal–Wallis test with Dunn’s multiple comparisons test (* *p* < 0.05, ** *p* < 0.01, *** *p* < 0.001). A phylogenetic tree of tested betacoronavirus spike sequences was generated in Geneious Prime version 2024.0.2.

## 3. Results

### 3.1. Design of Scaffolded RBD Constructs

**Figure 1:**
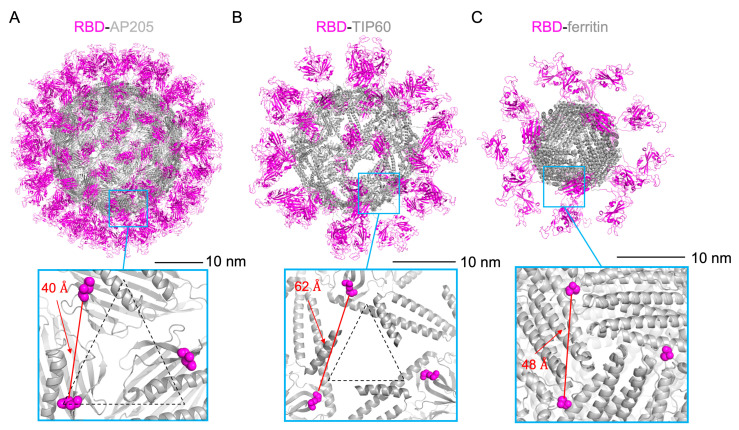
Visualization and structural analysis of scaffolded RBD constructs. (**A**) Full model of BA.4/5 RBD fused to AP205, a 180-mer phage capsid protein. Closer inspection of three-fold axis of symmetry in RBD-AP205 model surface suggests attachment of RBDs at distance of 40 Å, which is a shorter distance than found in ferritin attachment points. (**B**) Full model of BA.4/5 RBD fused to TIP60, a designed 60-mer nanoparticle assembly. Closer inspection of three-fold axis of symmetry in RBD-TIP60 model surface suggests attachment of RBDs at distance of 62 Å, which is a longer distance than found in ferritin attachment points. (**C**) Full model of BA.4/5 RBD fused to *H. pylori* ferritin with a linker from bullfrog ferritin at the N-terminus. Models are shown with scaffolds colored gray and RBDs colored magenta. Models are also sized roughly to scale, demonstrating differences in diameter of expected assemblies. In three-fold axis sections of (**A**–**C**), N-termini of AP205, TIP60, and ferritin scaffolds are shown as sticks colored magenta, with the distance measurement shown as a red line. The distance between ferritin attachment points within the corresponding three-fold axis (48 Å) is represented by a black triangle with dashed lines in (**A**,**B**) as a reference. The measurement wizard in PyMOL was used to estimate both the distance between N-terminal attachment points and the diameter of full model assemblies.

Our design approach began by reviewing available structural data [84] to select assemblies based on defined scaffold properties, including monomer size (<300 amino acids, to accommodate encoding by mRNA), sequence origin (non-eukaryotic or synthetic to avoid inducing autoreactivity in humans), and exposure of N-termini to the surface (to allow for antigen fusion). This selection process allowed for scaffolds known to form larger particles and present more antigen copies than ferritin, while also facilitating direct comparisons of the immunogenicity of RBD presentation following mRNA delivery. As a result, the large, self-assembling nanoparticles AP205 and TIP60 were selected to present RBD copies. AP205 is a phage capsid protein known to infect *Acinetobacter* bacteria [85] and forms a large 180-mer assembly [59], theoretically allowing for 180 copies of RBD to be presented on its surface if fused by a flexible linker. TIP60 is a designed nanoparticle formed from a two-fold assembly (coiled-coil domain of Myosin X) and a five-fold assembly (putative Sm-like protein) strategically fused together to adopt a 60-mer assembly [60,86], with the ability to display 60 copies of RBD. The functions of these domains in TIP60 involve antiparallel dimer formation and RNA binding, differing from the role of AP205 capsid in attachment and genome storage. To visualize these designs, the structure of the BA.4/5 variant of SARS-CoV-2 RBD was modeled onto each self-assembling protein, incorporating the flexible glycine–serine linker previously used for RBD-ferritin [57], with predicted structural models of each assembly shown in Figure 1. In addition to greatly exceeding the number of 24 RBDs displayed on ferritin, both RBD-AP205 and RBD-TIP60 adopt a larger particle size (~30–35 nm in diameter) (Figure 1A,B) than RBD-ferritin (~15–20 nm). As demonstrated elsewhere, this increased size may give the immune system more opportunities for antigen recognition, B-cell crosslinking, and robust neutralizing responses [29,52,87]. Though RBD-AP205 and RBD-TIP60 were predicted to be similar in size, there is a clear difference in structure, as the smaller number of copies in TIP60 leads to a more porous assembly. The three constructs also provided subtle differences in the spacing of RBD display, as suggested by the estimated distances between N-terminal attachment points in a common 3-fold axis of symmetry. With 40 Å between RBDs, display on RBD-AP205 represented a denser array than RBD-ferritin (48 Å) (Figure 1A), while RBDs on RBD-TIP60 were displayed in a sparser array (62 Å) (Figure 1B). 

### 3.2. Characterization of Scaffolded RBD Constructs

For initial characterization, RBD-AP205 and RBD-TIP60 were cloned and expressed in HEK293F cells along with RBD-ferritin to evaluate secretion of these assemblies to supernatant following transient transfection. The supernatant was first analyzed by western blot under denaturing conditions; here, samples from all three scaffolded RBD constructs were recognized by an anti-RBD monoclonal antibody, showing bands corresponding to the expected sizes (~40–50 kDa) of RBD-ferritin/RBD-TIP60/RBD-AP205 monomers (Figure S2A). While RBD-AP205 showed expression comparable to RBD-ferritin, RBD-TIP60 expression was noticeably lower and more supernatant volume was needed for appropriate detection. RBD-TIP60 under denaturing conditions also showed an additional band approximately the size of a trimer (~135 kDa). Expression and secretion of RBD-AP205 and RBD-TIP60 was also confirmed by sandwich ELISA using anti-RBD monoclonal antibodies (Figure S2B). The endpoint titers were higher than RBD-ferritin, though the titer increase was more pronounced for RBD-AP205 than RBD-TIP60 (Figure S2B). Since these designed constructs were expected to spontaneously form large assemblies, we sought to understand the distribution and proportion of species in each expressed protein. Supernatant of scaffolded RBDs from transient transfection was concentrated and run in Tris-Acetate gels under native conditions. The results show one or more high molecular weight species for RBD-AP205, RBD-TIP60, and RBD-ferritin, suggesting that a portion of the sample can form large assemblies (>1 MDa). RBD-AP205 and RBD-ferritin showed both a clear band at the top of the gel and a smear at or above 460 kDa in western blot (Figure S3A), exhibiting sample heterogeneity and potentially highlighting the dynamic process of particle assembly. RBD-TIP60 again showed lower expression, but a band at the top of the gel was detected. Yet RBD-TIP60 was not homogenous, as an additional band approximately the size of a pentameric assembly (~250 kDa) was found when more supernatant was loaded (Figure S3A). When stained with coomassie blue (Figure S3B), each supernatant showed a band at or above 1236 kDa, suggesting that the highest molecular weight bands detected by western blot approximated the expected molecular weight of each full assembly. Though high molecular weight species greater than 1 MDa were only a portion of a heterogenous sample, their presence suggested that each design can self-assemble as intended and at comparable rates.

### 3.3. Immunogenicity of Scaffolded RBD Constructs

**Figure 2:**
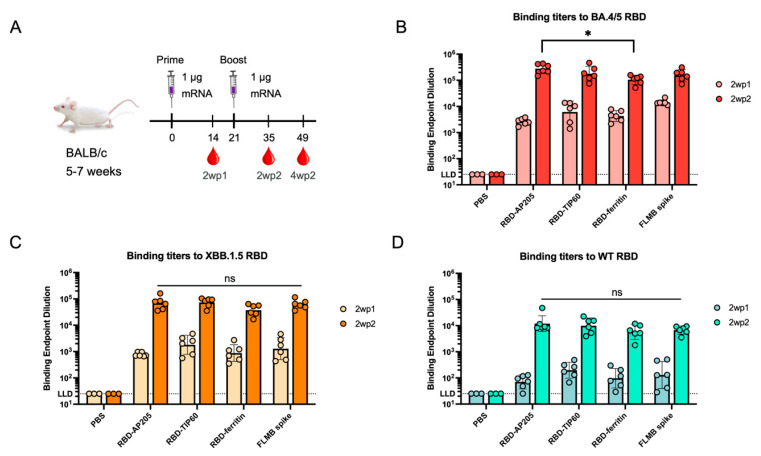
Endpoint titers of mouse sera immunized with RBD-scaffold designs to SARS-CoV-2 variant RBDs. (**A**) Study design with 5–7-week-old BALB/c mice, consisting of a prime and boost with 1 μg mRNA-LNP formulations three weeks apart. Mouse sera were collected two weeks post the 1st dose (2wp1), two weeks post the 2nd dose (2wp2), and four weeks post the 2nd dose (4wp2). All immunizations included six mice per group except the PBS group (three mice). Numbers under the gray line indicate the day since the start of the study for all immunization and bleed timepoints. Binding titers of sera in ELISA to (**B**) BA.4/5 RBD, (**C**) XBB.1.5 RBD, and (**D**) WT RBD shown for the 2wp1 and 2wp2 timepoints. A full-length, membrane-bound (FLMB) spike was used as a secondary control for assessing titers from immunizations. Endpoint dilutions were calculated as a geometric mean, with geometric standard deviation represented as error bars. The dots in each bar graph indicate the titers for individual mice in the study. Any titer that did not reach the lower limit of detection (LLD) was assigned the LLD value for illustrative purposes. These results are representative of two independent animal experiments. Tests for significance of 2wp2 titers determined by the Kruskal–Wallis test with Dunn’s multiple comparisons test (* *p* < 0.05, ns = not significant).

**Figure 3:**
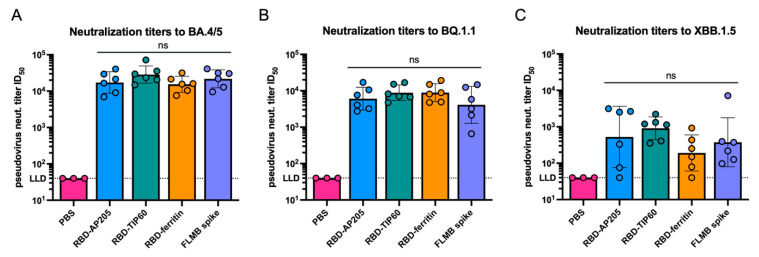
Neutralization titers of mouse sera immunized with RBD-scaffold designs to SARS-CoV-2 Omicron variants. Induction of neutralizing titers by 2wp2 sera to (**A**) BA.4/5, (**B**) BQ.1.1, and (**C**) XBB.1.5 pseudovirus was measured as ID_50_ values. A full-length, membrane-bound (FLMB) spike was used as a secondary control for assessing titers from immunizations. Endpoint dilutions were calculated as a geometric mean, with geometric standard deviation represented as error bars. The dots in each bar graph indicate the titers for individual mice in the study. Any titer that did not reach the lower limit of detection (LLD) was assigned the LLD value for illustrative purposes. These results are representative of two independent animal experiments. Tests for significance of 2wp2 data were determined by the Kruskal-Wallis test with Dunn’s multiple comparisons test (ns = not significant).

Having demonstrated the expression and antigenicity of these constructs, we next assessed the immunogenicity of these designs when delivered as messenger RNA (mRNA) formulated with lipid nanoparticles (LNPs) in vivo. RBD-AP205, RBD-TIP60, and RBD-ferritin were cloned into an mRNA vector, produced as mRNA through in vitro transcription (IVT). Each mRNA solution was then encapsulated in LNPs through mixing with ionizable lipids in a microfluidic system. Formulation of mRNA with LNPs resulted in particles of uniform size, low polydispersity, and high encapsulation efficiency as determined by dynamic light scattering (DLS) and RiboGreen assay, and low endotoxin levels (~0.04 EU per injection) (Table S1). These samples were used for homologous intramuscular immunization at a dose of 1 μg mRNA in naïve BALB/c mice, given three weeks apart (Figure 2A). mRNA-LNP encoding a full-length membrane-bound (FLMB) BA.4 variant spike trimer was used as a positive control during immunization. Mouse sera were obtained for analysis at two weeks following the 1st dose (2wp1) and the 2nd dose (2wp2). All constructs induced high serum endpoint titers (10^5^–10^6^) in ELISA to homologous BA.4/5 RBD by 2wp2, showing that RBD-AP205 and RBD-TIP60 can match the immunogenicity not just of a commonly used scaffold displaying multiple RBDs, but a construct mimicking licensed SARS-CoV-2 mRNA vaccines (Figure 2B). Interestingly, RBD-AP205 titers at 2wp2 were found to be significantly higher than RBD-ferritin titers (*p* < 0.05), suggesting an immunogenicity advantage of AP205 display. Endpoint titers in 2wp2 sera to heterologous strains (XBB.1.5 RBD and ancestral WT RBD) showed no significant differences across constructs, suggesting that RBD-AP205 and RBD-TIP60 induce cross-reactive antibody responses comparable to the benchmarks (Figure 2C,D). Here, reduced binding titers to XBB.1.5 (~10^5^) and WT (~10^4^) were expected given the sequence differences. Next, we assessed the function of serum responses elicited by each construct using a pseudovirus neutralization assay. Following two immunizations, RBD-AP205 and RBD-TIP60 elicited robust neutralizing titers (ID_50_ ~10^4^) to BA.4/5 (Figure 3A), demonstrating these scaffolds can present neutralizing RBD epitopes. These neutralizing titers were comparable to RBD-ferritin and FLMB spike titers to the vaccine-matched variant, BA.4/5, and this trend was maintained for BQ.1.1 and XBB.1.5 variants (Figure 3B,C). RBD-TIP60 neutralization titers trended higher than RBD-ferritin, particularly against XBB.1.5, but the difference was not significant.

### 3.4. Comparison of Antibody Reactivity Breadth to Other Coronaviruses

**Figure 4:**
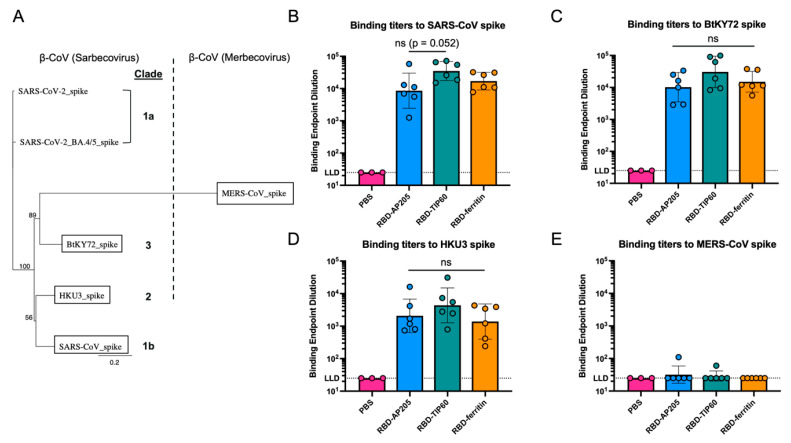
Breadth of immunogenicity of mouse sera immunized with RBD-scaffold designs to sarbecovirus clades and MERS-CoV. (**A**) Phylogenetic tree of utilized coronavirus spike sequences. WT and BA.4/5 spike sequences included as representatives of sarbecovirus clade 1a. Sarbecovirus clade number for other betacoronavirus spike proteins is included next to the corresponding sequence in bold. A vertical dashed line was incorporated to highlight the genetic distance between sarbecovirus sequences and MERS-CoV. The phylogenetic tree was generated in Geneious Prime. Bootstrapping values and a branch length key (0.2) were included automatically. Endpoint titers of 4wp2 mouse sera immunized with RBD-scaffold designs were evaluated in ELISA to (**B**) SARS-CoV spike, (**C**) BtKY72 spike, (**D**) HKU3 spike, and (**E**) MERS-CoV spike at the four weeks post 2nd dose (4wp2) timepoint. Endpoint dilutions were calculated as a geometric mean, with geometric standard deviation represented as error bars. The dots in each bar graph indicate the titers for individual mice in the study. Any titer that did not reach the lower limit of detection (LLD) was assigned the LLD value for illustrative purposes. Tests for significance of 4wp2 data were determined by the Kruskal–Wallis test with Dunn’s multiple comparisons test (ns = not significant).

We next extended the analysis of vaccine breadth to assess activity against more distantly related coronaviruses. Here we selected representatives of sarbecovirus clade 1b (SARS-CoV), clade 2 (bat coronavirus HKU3), and clade 3 (bat coronavirus BtKY72), which have spike proteins more genetically distant than the differences between SARS-CoV-2 variants [88,89,90,91,92] (Figure 4A). As a negative control, Middle Eastern respiratory syndrome coronavirus (MERS-CoV), was added as its cross-reactivity with SARS-CoV-2 is limited to highly conserved epitopes in S2 [93,94,95]. The 4wp2 sera from mice immunized with scaffolded RBD constructs showed appreciable binding titers to each sarbecovirus spike protein in ELISA (10^3^–10^4^) (Figure 4B–D). Binding titers were not found for any group to MERS-CoV, as expected [96,97] (Figure 4E). RBD-TIP60 titers trended higher than RBD-ferritin and RBD-AP205 to all tested sarbecoviruses, with a 2–3-fold increase over RBD-ferritin titers depending on the spike protein. The largest difference in titers occurred between RBD-TIP60 and RBD-AP205 to SARS-CoV, though this trend was not found to be significant (*p *= 0.052; Figure 4B). RBD-TIP60 trended toward eliciting the broadest responses across sarbecoviruses, suggesting that TIP60 presentation could display conserved RBD epitopes more readily.

## 4. Discussion

*H. pylori* ferritin has previously been used to display multimers of the SARS-CoV-2 RBD that boost neutralizing responses to a critical component of SARS-CoV-2 infection [57]. Here, we investigated if RBD presentation on AP205 or TIP60 assemblies could elicit similar or superior antibody responses when delivered as mRNA-LNPs. This concept was built from the full-length membrane-bound (FLMB) spike protein used in licensed vaccines [2,3] while potentially improving antigen display through multimerization. Though antibody binding and neutralization titers were largely comparable, RBD-AP205 induced significantly higher titers to the homologous BA.4/5 spike than RBD-ferritin in mouse sera. RBD-TIP60 showed binding titers that trended higher against a broad panel of sarbecoviruses. These results suggest that scaffolding with AP205 or TIP60 could produce useful immunogens resembling ferritin display, while potentially offering distinct features that can become useful in other vaccine applications.

Our work here raises several questions that warrant further investigation. Though the antigenicity and immunogenicity of RBD-AP205 and RBD-TIP60 designs appear promising, multiple paths for optimization should be explored. First, both scaffold sequences were unmodified, despite putative N-linked glycosylation sites, and the linker length of six amino acids used for RBD-ferritin [57,98] was not changed. The two predicted glycosylation sites in AP205 (N45, N75) and one predicted site in TIP60 (N18) could decrease the efficiency of assembly by introducing new contacts with the antigen and other scaffold monomers, though only the residue on TIP60 is close to the N-terminus used for fusion. All these components may contribute to the particle heterogeneity observed in this study. Some properties have already been altered for ferritin and other scaffolds [99,100,101], and AP205/TIP60 may benefit from similar modifications. Given its lower expression relative to RBD-ferritin and RBD-AP205, RBD-TIP60 may be limited by properties of the scaffold sequence, but may be improved with further antigen or scaffold optimization. More specifically, future work could alter linker length, linker rigidity [102,103,104], or the presence of N-linked glycosylation sites [101,105]. Optimizing these properties in RBD-AP205 and RBD-TIP60 may improve expression, yield, and stability by ensuring adequate spacing between subunits and increasing the efficiency and strength of assembly. Second, the BA.4/5 RBD sequence was not modified to maintain variant immunogenicity for all constructs. Though this design served the primary goal of comparing immunogenicity of RBD display by scaffold, properties of the unmodified sequence likely contributed to suboptimal yields and heterogeneity upon expression, limiting characterization efforts. BA.4/5 RBD has shown reduced purification yields compared to ancestral strains [106], and challenges in the purification of unmodified RBD-presenting nanoparticles have been documented in several previous studies [45,57]. Though the reasons for decreased yield are not clear, BA.4/5 RBD contains several mutations that increase hydrophobicity (S371F/S373P/S375F/T376A) [58], which could alter RBD properties in a way that impacts the stability or solubility of multimeric assembly. Future research could incorporate several mutations to hydrophobic residues in RBD (Rpk9 to fill the linoleic acid binding pocket, mutate residues 518–520 to N-linked glycosylation site) that have substantially increased the yield of monomeric and multimerized RBD [57,107,108]. Overall, future research should explore the incorporation of mutations that can boost construct yield and stability, especially for characterization efforts such as structure determination or antibody affinity measurements. These efforts can also help elucidate how determinants of antigenicity and immunogenicity for RBD constructs may differ, as RBD-AP205 endpoint titers in ELISA suggested superior antigenicity, yet immunogenicity was largely comparable to RBD-TIP60 and RBD-ferritin.

Due to their inherently weak immunogenicity as monomers, subunit antigens will benefit from multimeric display for optimal immunogenicity [29,30,31,32]. This research presents new examples of multimers that, when delivered as mRNA, can enhance the immunogenicity of a subunit antigen through self-assembly. In this approach, we found that describing the characterization of nanoparticle assembly in vivo to be difficult to address directly. As a result, we expect a mixture of assembled, partially assembled, and unassembled particles produced in vivo after mRNA delivery, marking a clear limitation in this study. However, the designs of scaffolded RBDs as mRNA compare favorably to similar constructs presented in the literature and highlight the benefits of the delivery platform. This result is evident for TIP60, which had not been used as a vaccine scaffold to date, though previous studies note its potential use as a carrier for other compounds [109,110]. AP205 has already been used as a platform for scaffolding SARS-CoV-2 RBD [111,112], with at least one construct tested in macaques [113] and a phase 1 clinical trial [114]. However, many of these studies use SpyTag/SpyCatcher technology [115] to express RBD and AP205 separately and conjugate after incubation, a multi-step process reliant on protein purification. But encoded as mRNA, RBD-AP205 confers benefits in speed and simplicity, compared to SpyTag/SpyCatcher conjugation. Previously, Liu and colleagues studied a vaccine derived from AP205 genetically fused to a SARS-CoV-2 antigen [116], though with some important differences to our work. That study did not evaluate mRNA, used only the receptor binding motif (RBM), and utilized dimerized AP205 subunits resulting in only 90 RBM copies per particle [116]. While our work is in general agreement with Liu and colleagues, here we designed a genetic fusion with the complete RBD to AP205 and demonstrated strong immunogenicity when delivered as mRNA.

Since AP205 and TIP60 presentation of RBD was largely comparable to ferritin, these assemblies may be considered in future vaccine designs as part of a scaffold “toolbox” to test alongside commonly used nanoparticles. Protein subunit presentation by these scaffolds could warrant further comparison with scaffolds such as lumazine synthase [45], I3-01 [108,117], and others previously engineered to present SARS-CoV-2 RBD [24,118]. Since each assembly exhibits a different spacing of N-terminal attachment points, further comparisons could offer insights on how relative accessibility of RBD epitopes may affect the breadth of antibody reactivity and potential application as mosaic particles with RBDs from multiple coronaviruses [35,43,119,120]. The increased spacing of attachment points did not correlate strongly with increased binding and neutralization titers in this study, but this concept should remain a focus for future studies. Spacing and accessibility could be adjusted within scaffolds through altered linker lengths or properties, as demonstrated by an N-terminal extension to ferritin for improved spacing of RBD [57] and other antigens [99,100]. Through this work, researchers may better understand the dynamics of antigen spacing, which suggests that scaffold assembly can be tailored to an antigen to maximize immunogenicity and neutralization breadth. This comparison can also extend beyond SARS-CoV-2 to other betacoronaviruses for pan-coronavirus vaccine design [121,122,123]. With additional scaffolds available for mRNA delivery, future research could help determine the optimal properties for presenting an antigen subunit of interest to maximize vaccine immunogenicity.

## 5. Conclusions

In this work, we sought to identify and test multimeric scaffolds for RBD presentation that could also be delivered as mRNA-LNPs. AP205 and TIP60 scaffolds were found to elicit immune responses that were comparable to the ferritin platform commonly used for antigen display. Through this direct comparison with a benchmark vaccine candidate, we established the utility of these multimeric scaffolds, especially in the context of mRNA delivery. As a result, AP205 and TIP60 can be considered alongside other frequently used platforms for antigen multimerization, a technique that has been shown to improve the immunogenicity of subunit antigens, expanding potential options for future vaccine design.

## Data Availability

All data are either presented in this manuscript or included as Supplementary Materials.

## Supplementary Materials

The following supporting information can be downloaded at: https://www.mdpi.com/article/10.3390/vaccines13080778/s1, Figure S1: Plasmid map for RBD-ferritin utilized for the cloning of RBD-AP205 and RBD-TIP60. Figure S2: RBD-scaffold constructs detected in the supernatant of transfections using Western Blot and ELISA. Figure S3: All scaffolded RBD constructs display high molecular weight species in NativePAGE gels. Table S1: Summary of physicochemical analysis of mRNA formulated with LNPs. Figures S4–S7: Uncropped images of western blot and Coomassie in Figures S2 and S3.
