# Supplementary material for: SARS-CoV-2 RBD Scaffolded by AP205 or TIP60 Nanoparticles and Delivered as mRNA Elicits Robust Neutralizing Antibody Responses
Source: Vaccines (Basel). 2025 Jul 22;13(8):778. doi: 10.3390/vaccines13080778 (PMC12389965; doi:10.3390/vaccines13080778)
Supplement: Supplementary file 1 [file vaccines-13-00778-s001.zip › vaccines-3696539-supplementary.pdf]

Supplementary Information

SARS-CoV-2 RBD scaffolded by AP205 or TIP60 nanoparticles and delivered as mRNA elicits robust neutralizing antibody responses

Johnathan D. Guest, Yi Zhang, Daniel Flores, Emily Atkins, Kuishu Ren, Yingyun Cai, Kim Rosenthal, Zimeng Wang, Kihwan Kim, Charles Chen, Richard Roque, Bei Cheng, Marianna Yanez Arteta, Liping Zhou, Jason Laliberte, and Joseph R. Francica

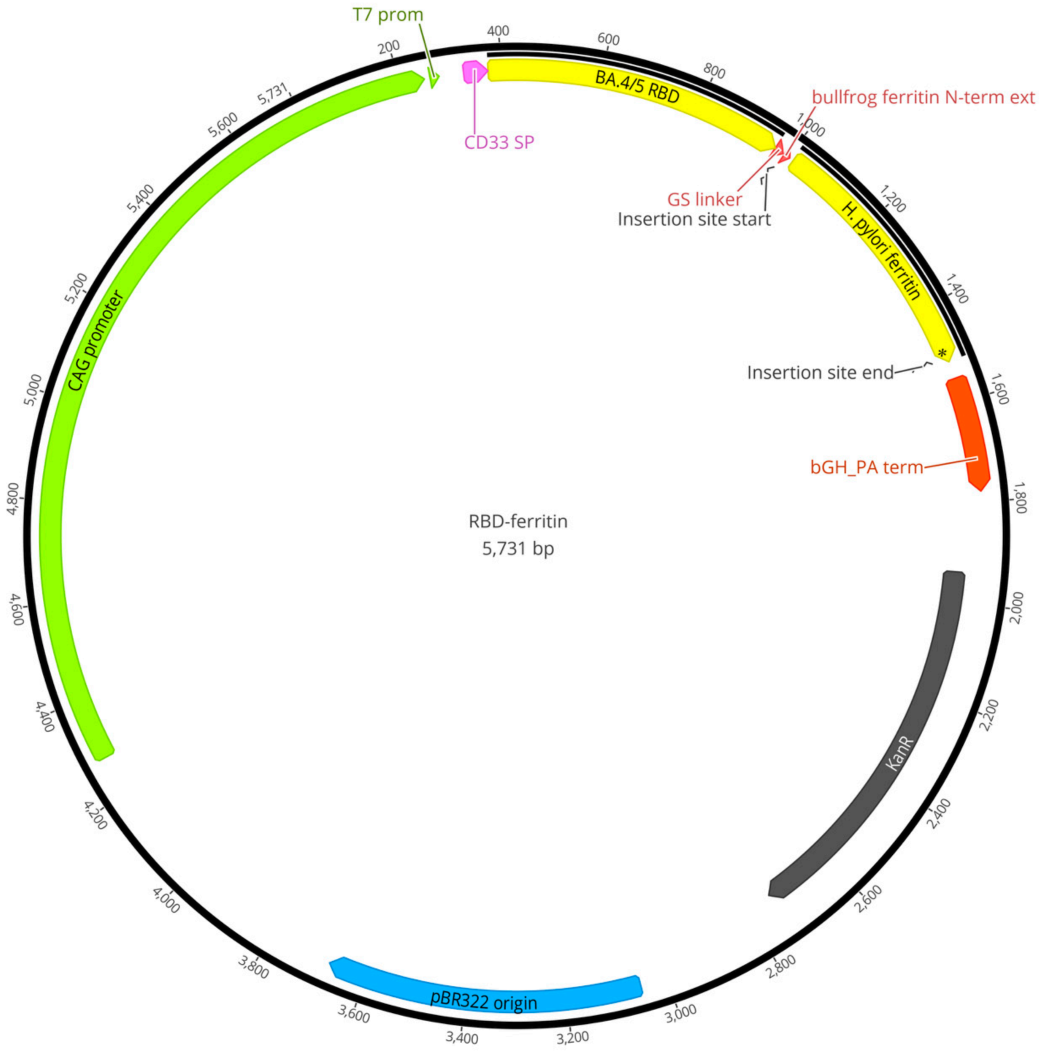

Figure S1: Plasmid map for RBD-ferritin utilized for cloning of RBD-AP205 and RBD-TIP60. The plasmid map for RBD-ferritin was exported from Geneious Prime and annotated to show properties relevant for cloning. The coding sequence of RBD-ferritin is highlighted in yellow, with red markers in the middle of the sequence indicating the location of the flexible glycine-serine linker (GS linker) and the N-terminal extension from bullfrog ferritin (bullfrog ferritin N-term ext). Also incorporated in the plasmid are the CAG promoter (in green), T7 RNA polymerase promoter sequence (T7 prom, in green), CD33 signal peptide (CD33 SP, in magenta), bgh polyadenylation signal sequence (bGH_PA term, in orange), kanamycin resistance gene (KanR, in gray), and pBR322 origin of replication (pBR322 origin, in blue). The gray lines labeled insertion site start and end indicate the location for where the ferritin sequence was removed and replaced by AP205 or TIP60 fragments through Gibson assembly.

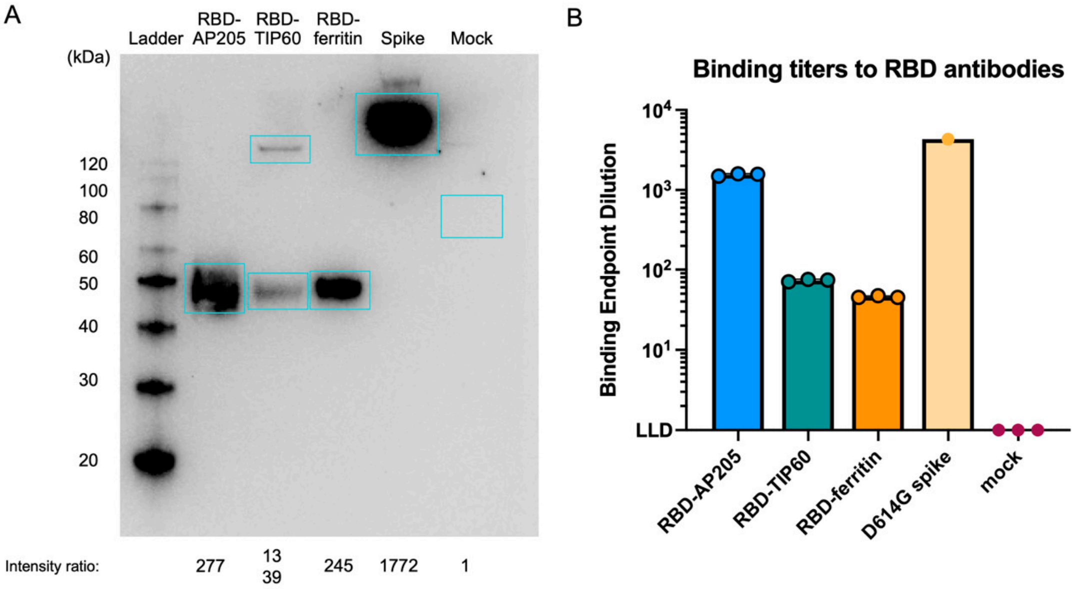

Figure S2: RBD-scaffold constructs detected in the supernatant of transfections using Western Blot and ELISA. (A) An anti-RBD monoclonal antibody detected RBD-scaffold constructs from respective concentrated supernatants under reducing conditions. Each supernatant sample displayed a band near the expected monomer size (40-50 kDa) according to MagicMark XP standard (ThermoFisher). To clearly visualize all monomer bands, different protein amounts of concentrated supernatant were loaded according to A280 values. 5 μg of RBD-TIP60 and Mock were loaded, along with 500 ng of RBD-AP205 and RBD-ferritin. 50 ng of purified SARS-CoV-2 WT spike protein was also loaded as a positive control. Intensity ratios of western blot bands were calculated in Image Lab by comparing the band intensity to a background volume, determining a relative volume quantity while adjusting for local background. Areas used for calculations are outlined in light blue. For RBD-TIP60, the ratio was 13 for the higher molecular weight band and 39 for the lower molecular weight band. (B) Harvested supernatant from RBD-AP205, RBD-TIP60, and RBD-ferritin expressions showed appreciable endpoint titers in a sandwich ELISA with anti-RBD antibodies. Endpoint dilutions were calculated as the geometric mean from three technical replicates. Any titer that did not reach the lower limit of detection (LLD) was assigned the LLD value for illustrative purposes. SARS-CoV-2 spike trimer (D614G spike) was included as a positive control recognized by both antibodies. Spike protein was serially diluted in one replicate starting at 3 μg/mL to assess binding endpoint dilution. Mock supernatant contained no transfection reagent or plasmid during transfection. ELISA plates were read by absorbance at 450 nm.

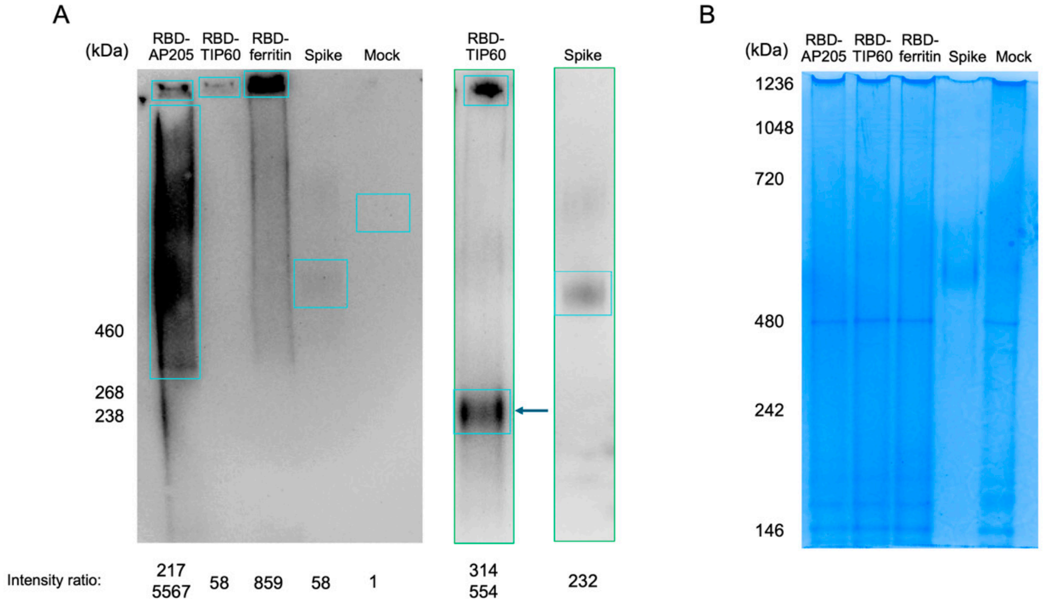

Figure S3: All scaffolded RBD constructs display high molecular weight species in NativePAGE gels. (A) Each RBD-scaffold design in concentrated supernatant were detected in one or more bands greater than 460 kDa in size by an anti-RBD monoclonal antibody. 3 µg of RBD-AP205, RBD-TIP60, RBD-ferritin, and mock supernatant was loaded, with volumes adjusted according to the measured A280 of total protein concentration. Intensity ratios of western blot bands were calculated in Image Lab by comparing the band intensity to a background volume, determining a relative volume quantity while adjusting for local background. Areas used for calculations are outlined in light blue. For RBD-AP205, the ratio was 217 for the higher molecular weight band and 5567 for the smeared band. Cropped gel images outlined in green are sections from the same blot, but are not from the same blot as the gel image with no outline. Loading more RBD-TIP60 (~15 µg) revealed an additional form of assembly under native conditions and is highlighted by a blue arrow. For RBD-TIP60, the ratio was 314 for the higher molecular weight band and 554 for the lower molecular weight band. 100 ng SARS-CoV-2 WT spike trimer was loaded in the gel image with no outline, while 50 ng spike trimer was loaded in the gel section outlined in green. (B) Concentrated supernatants of scaffolded RBD constructs and mock transfection in native conditions showed a similar pattern of bands when stained with coomassie blue. 6 µg of RBD-AP205, RBD-TIP60, RBD-ferritin, and mock supernatant was loaded, with volumes adjusted according to measured A280. 1 µg spike trimer was also loaded as a positive control. Mock supernatant contained no transfection reagent or plasmid during transfection. The image was colored coomassie blue with Image Lab software.

Table S1: Summary of physicochemical analysis of mRNA formulated with LNPs

	Size (nm)		PDI[1]		EE (%)[2]		Endotoxin test	
mRNA	Average	Std[3]	Average	Std	Average	Std	Stock (EU/mL)	Per injection (EU)[4]
RBD-AP205	84.85	1.78	0.119	0.007	93.78	0.17	7.36	0.0368
RBD-TIP60	83.63	1.56	0.108	0.014	94.34	0.07	8.02	0.0401
RBD-ferritin	85.61	2.17	0.112	0.011	93.61	0.61	7.74	0.0387
FLMB spike	87.34	1.81	0.122	0.005	93.52	0.19	8.62	0.0431

[1]Polydispersity index, as reported from dynamic light scattering (DLS)

[2]Encapsulation efficiency of mRNA into LNPs (out of 100%)

[3]Standard deviation (N=3)

[4]Endotoxin units (EU) delivered to each mouse per injection. EU was calculated based on stock concentration multiplied by the volume of stock solution (5 µL) delivered to each mouse. 5 µL stock solution (equal to 1 µg mRNA-LNP) was diluted in sterile PBS to 50 µL volume for preparation prior to injection.

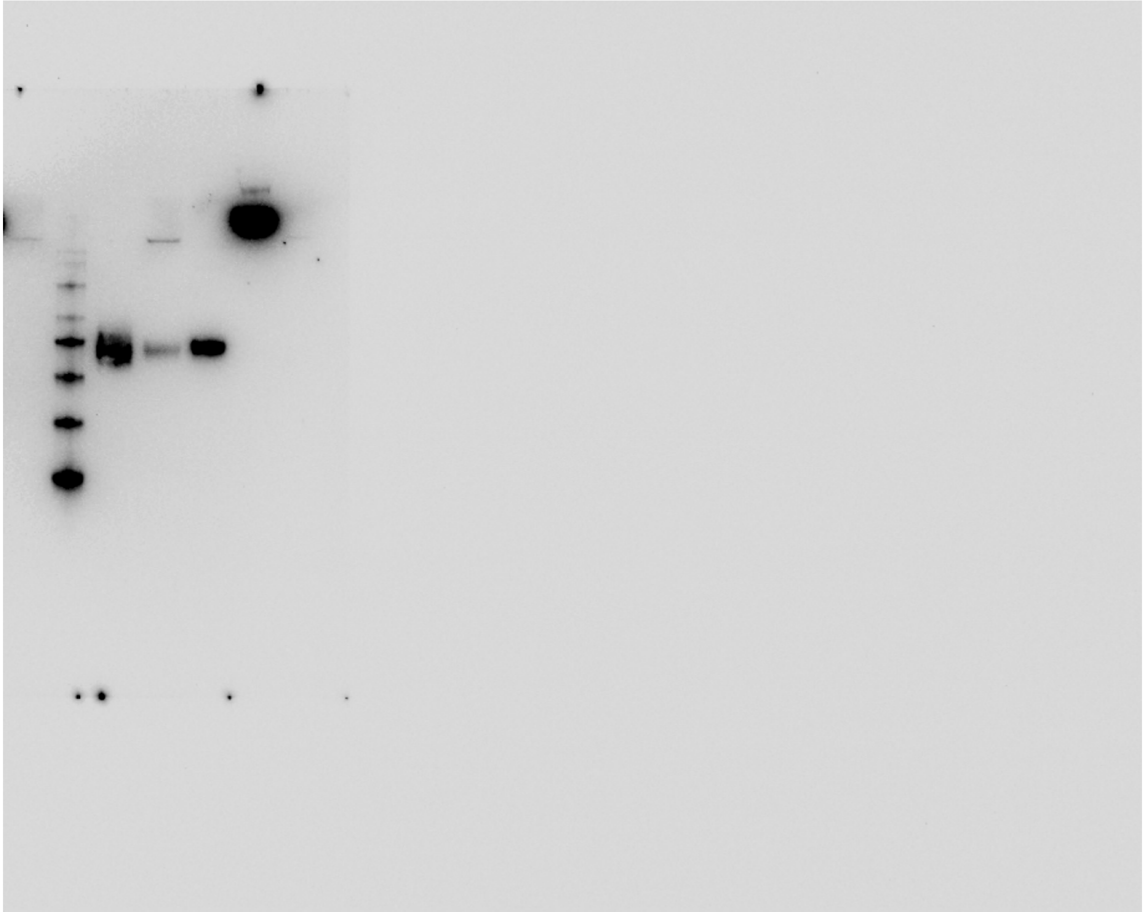

Figure S4: Uncropped image for western blot used in Figure S2A

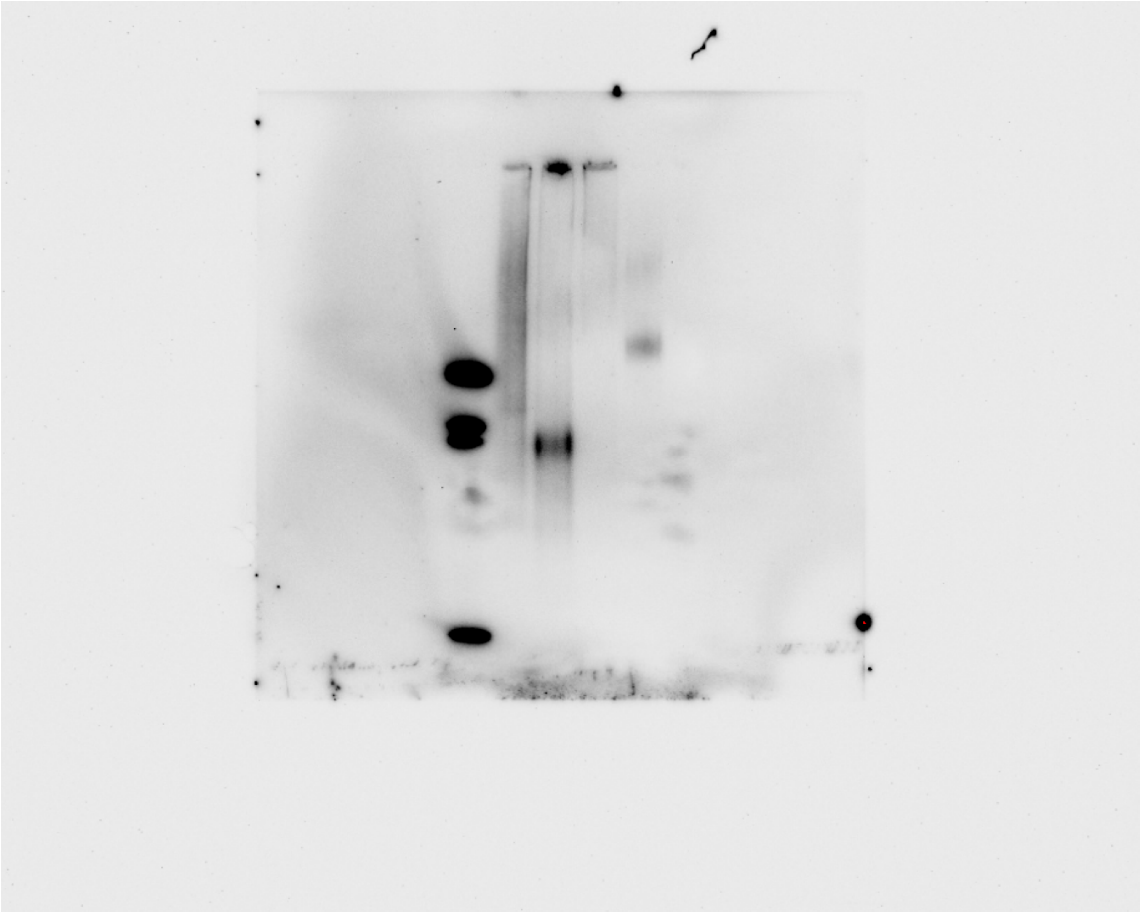

Figure S5: Uncropped image for western blot used in Figure S3A (sections with green outline)

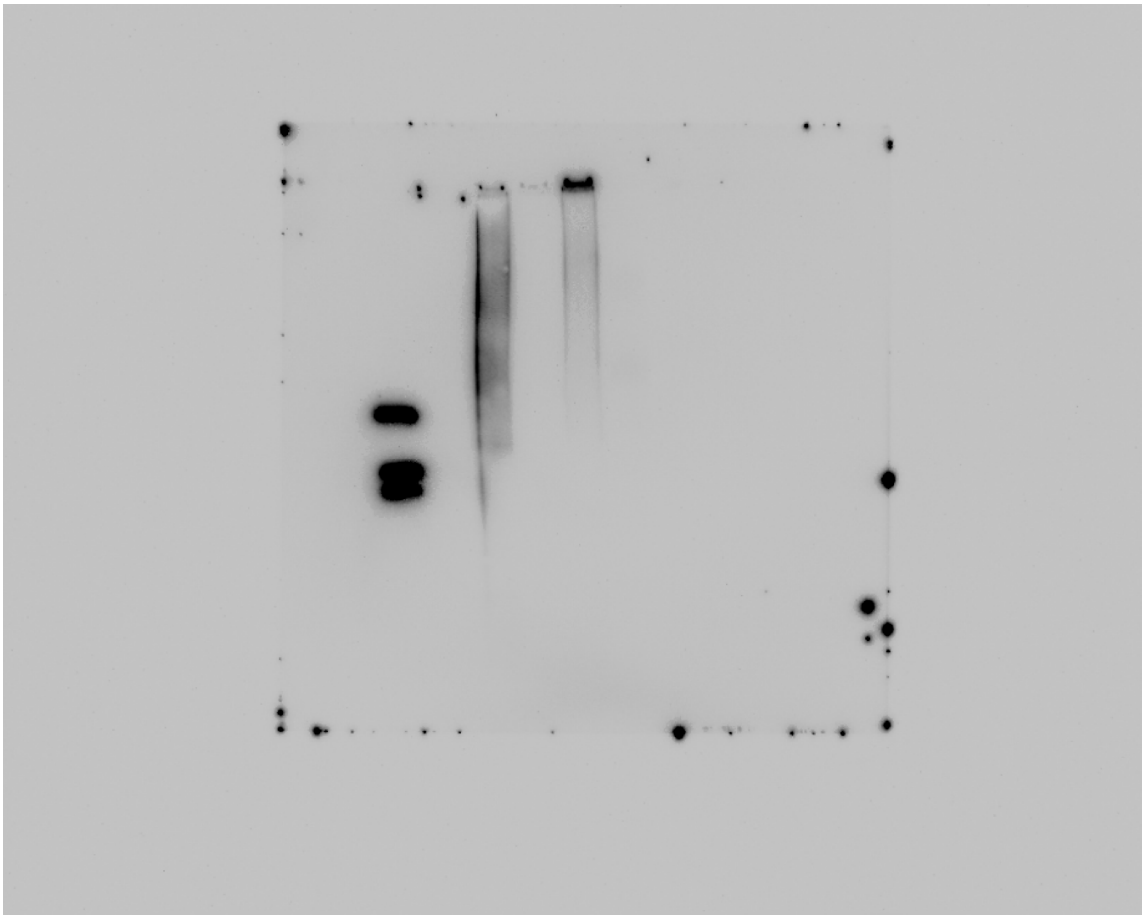

Figure S6: Uncropped image for western blot used in Figure S3A (image without green outline) – version in Figure S3A imaged without ladder in frame

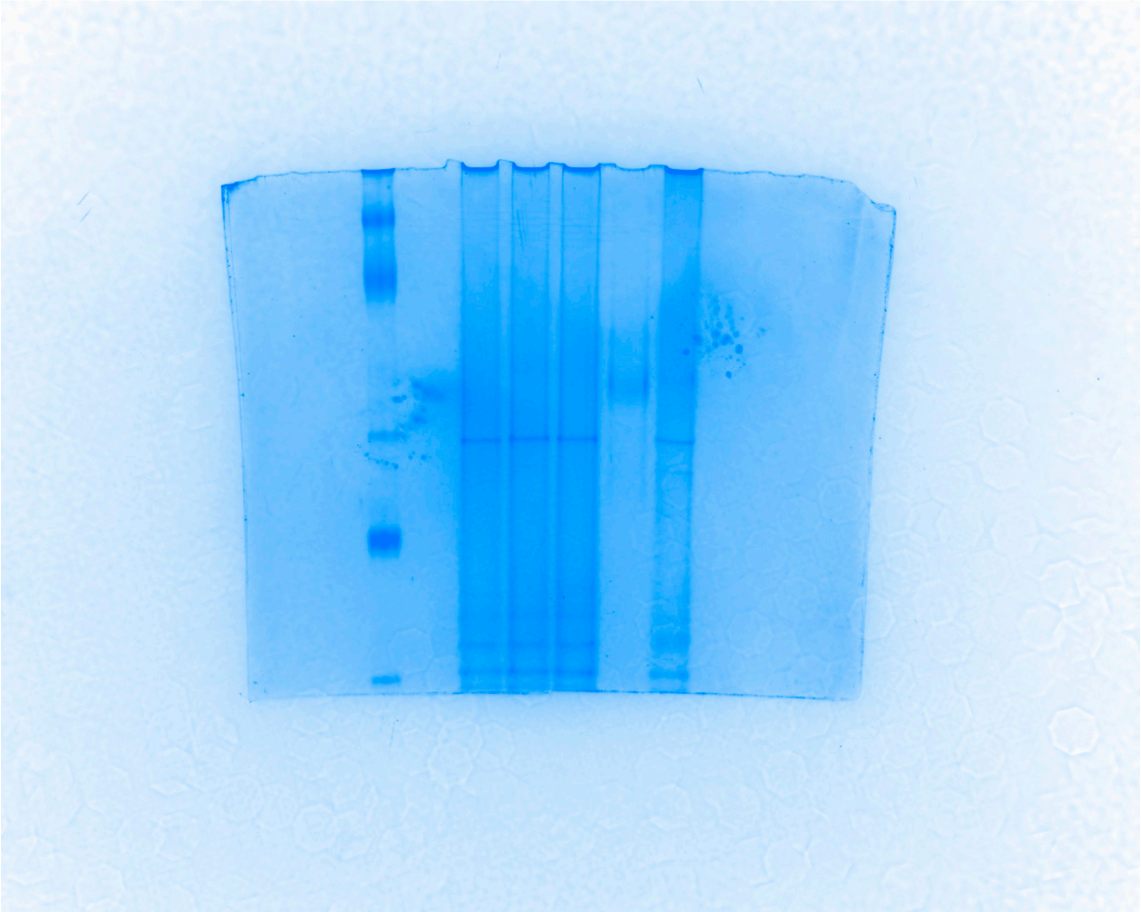

Figure S7: Uncropped image for coomassie blue native gel in Figure S3B
